# Draft Genome Sequence of Goose Dicistrovirus

**DOI:** 10.1128/genomeA.00068-16

**Published:** 2016-03-03

**Authors:** Alexander L. Greninger, Keith R. Jerome

**Affiliations:** Department of Laboratory Medicine, University of Washington, Seattle, Washington, USA

## Abstract

We report the draft genome sequence of goose dicistrovirus assembled from the filtered feces of a Canadian goose from South Lake Union in Seattle, Washington. The 9.1-kb dicistronic RNA virus falls within the family *Dicistroviridae*; however, it shares <33% translated amino acid sequence within the nonstructural open reading frame (ORF) from aparavirus or cripavirus.

## GENOME ANNOUNCEMENT

Canadian geese (*Branta canadensis*) are well known for their prolific production of feces, depositing up to one dropping per minute and up to 1.5 lb per day (1). This fact combined with their growing populations have raised concerns over the relation of the goose population to public health. Goose feces are known to contain a number of pathogens that infect humans and affect surface waters (2–4). Exploration of the viruses of Canadian geese has led to the finding of Newcastle disease virus, influenza virus, and bornavirus (1, 5, 6).

In this study, we sequenced filtrate of a singular goose dropping from Lake Union Park in Seattle, Washington. One gram of goose feces was mixed 1:10 (weight/volume) in phosphate-buffered saline (PBS), vortexed, and syringe-filtered through a 0.8-µm filter (Millipore). RNA from 1 mL of filtrate was extracted using the Zymo RNA miniprep kit using on-column DNase treatment. Sequencing library preparation from cDNA was performed using Nextera XT with 15 cycles of PCR amplification as described previously (7, 8). A total of 3.77 million paired-end 2- × 300-bp sequencing reads were *de novo* assembled using Abyss (9).

A 9,130-nucleotide contig was assembled that aligned by blastx to members of the *Dicistroviridae* family. The contig had 55× coverage and contained two large open reading frames (ORFs) of 5,481 and 2,577 nucleotides, along with a 352-nucleotide intergenic region. The first ORF contained conserved domains for RNA helicase, C3G peptidase, and RNA-dependent RNA polymerase, while the second ORF contained conserved domains for picornavirus and cricket paralysis virus (CRPV) capsid proteins. The putative translated nonstructural protein gene sequence best aligned with 33% amino acid identity to cricket paralysis virus (AKA63263) while the capsid protein best aligned with 35% amino acid identity to Drosophila C virus (NP_044946). The translated nonstructural gene was also 32% identical by amino acid to the acute bee paralysis virus polymerase protein, indicating substantial divergence from both cripavirus and asparaviruses of the *Discistroviridae* family.

The genome likely encodes an insect virus that is only found associated with goose feces, rather than a bona fide goose virus. Of note, a 4,177-nucleotide contig was also assembled that aligned with 96% identity by nucleotide to subterranean clover mottle virus isolate MJ (AY376453) (10). Further study will be required to decipher the true host of goose dicistrovirus, as well as characterize unique coding aspects of the dicistrovirus such as the intergenic region internal ribosome binding sequence.

### Nucleotide sequence accession number.

The GenBank accession number for goose dicistrovirus is KT873140.
